# The oral commensal *Streptococcus mitis* activates the aryl hydrocarbon receptor in human oral epithelial cells

**DOI:** 10.1038/ijos.2017.17

**Published:** 2017-06-16

**Authors:** Stian A Engen, Gro H Rørvik, Olav Schreurs, Inger JS Blix, Karl Schenck

**Affiliations:** 1 Department of Oral Biology, Faculty of Dentistry, University of Oslo, Oslo, Norway; 2 Institute of Clinical Dentistry, Faculty of Dentistry, University of Oslo, Oslo, Norway

**Keywords:** aryl hydrocarbon receptor, commensal, inflammation, oral epithelium, prostaglandin E2, *Streptococcus*

## Abstract

*Streptococcus mitis (S. mitis)* is a pioneer commensal bacterial species colonizing many of the surfaces of the oral cavity in healthy individuals. Yet, not much information is available regarding its interaction with the host. We used examination of its transcriptional regulation in oral keratinocytes to elucidate some of its potential roles in the oral cavity. Transcription factor analysis of oral keratinocytes predicted *S. mitis*-mediated activation of aryl hydrocarbon receptor (AhR). Activation and functionality of AhR was confirmed through nuclear translocation determined by immunofluorescence microscopy and real-time polymerase chain reaction with reverse transcription analysis of *CYP1A1*, the hallmark gene for AhR activation. Addition of *Streptococcus mutans* or *Streptococcus gordonii* did not induce *CYP1A1* transcription in the keratinocyte cultures. Introduction of an AhR-specific inhibitor revealed that *S. mitis*-mediated transcription of *CXCL2* and *CXCL8* was regulated by AhR. Elevated levels of prostaglandin E2 (enzyme-linked immunosorbent assay) in supernatants from *S. mitis*-treated oral epithelial cells were also attenuated by inhibition of AhR activity. The observed AhR-regulated activities point to a contribution of *S. mitis* in the regulation of inflammatory responses and thereby to wound healing in the oral cavity. The concept that the oral commensal microbiota can induce AhR activation is important, also in view of the role that AhR has in modulation of T-cell differentiation and as an anti-inflammatory factor in macrophages.

## Introduction

The immune system and other organs depend on successful, mutualistic interaction with the commensal microbiota for normal development and function. Animals born and raised in the absence of microorganisms suffer from severe local and systemic immune deficiencies.^1^ The commensal microbiota protects the host from pathogens either by directly targeting the colonizing pathogens or through indirect mechanisms supporting the immune system and modulating the host cellular responses to pathogens.^2, 3^ As the antigenic content (like environmental antigens and commensal microorganisms) differ by their anatomical location, anatomical and physiological characteristics also change, for example, the multilayered protective oral epithelium compared to the monolayer of the small intestine that absorb food nutrients. The enduring presence of the indigenous microflora by the immune system may prove beneficial in setting the tone of the immune defense and keeping it alert.

Complex communities of microorganisms colonize all niches of the human body and the oral cavity is no exception. More than 700 bacterial species can be found at this site^4, 5^ and a healthy oral microbiome may consist of more than 200 species.^6^ The commensal species *Streptococcus mitis (S. mitis)* is a predominant pioneer colonizer of the oral cavity from early infancy and throughout life^7^ and is thought to form the basis for oral biofilms by supplying adherence sites for secondary colonizers.^8, 9^ The precise reasons for *S. mitis* commensalism are as yet unclear, but some known qualities can contribute to its persistence in the oral cavity. *S. mitis* produces an IgA1 protease that may aid in the species’ colonization by deflecting elimination by the secretory immune system.^10^ The genome of *S. mitis* contains several sequences that can code for putative adhesins.^11^ Another feature that could account for commensalism in *S. mitis* is that the species is physiologically and antigenically diverse, properties that could compensate for elimination by host immune defenses and promote re-colonization or transfer to a new habitat.^12^

As we have shown previously, the presence of *S. mitis* is readily recognized by oral keratinocytes in culture and triggers transcription of the chemokines CXCL2 and CXCL8.^13^ The aim of the present study was to use investigation of the transcriptional regulation in oral keratinocytes exposed to *S. mitis,* to find relevant regulators that are activated by *S. mitis*. Among several other factors, we found that the transcription factor aryl hydrocarbon receptor (AhR) was activated and this was further examined.

## Materials and methods

### Bacterial strains

Overnight cultures of *S. mitis* CCUG 31611 T were prepared from glycerol stocks by inoculating 10 μL of stock in 10 mL fresh tryptone soy broth (TSB) (ThermoFisher, Waltham, MA, USA) and incubated at 37 °C and 5% CO_2_ saturation. Four milliliter of overnight cultures were diluted in 25 mL fresh, pre-warmed (37 °C) TSB and grown to OD_600_ of 0.460 (6 × 10^7^ bacteria per mL). The bacteria were pelleted at 10 000*g* for 10 min and resuspended in reduced keratinocyte serum-free medium (K-SFM) (ThermoFisher, Waltham, MA, USA) (without supplements and antibiotics) to a concentration of 3 × 10^7^ bacteria per mL. For preparation of bacterial lysates, a mid-log phase bacterial suspension (OD_600_ of 0.6) was pelleted at 10 000*g* for 10 min and resuspended in 800 μL phosphate-buffered saline (PBS) (Sigma-Aldrich, St Louis, MO, USA) before three freeze-thaw cycles of 30 min at −150 °C followed by 10 min at 37 °C. After the last cycle, the suspension was cleared by centrifugation at 13 000*g* for 30 min and the protein quantity of the supernatant determined (Bio-Rad, Hercules, CA, USA) before freezing at −20 °C for later use. The bacterial cell extract was not further defined and contained uncharacterized factors.

*Streptococcus gordonii (S. gordonii)* CCUG 25608 T and *Streptococcus mutans (S. mutans)* ATCC 700610 were grown as described for whole cell *S. mitis*.

### Human oral keratinocytes

The study was approved by the Regional Ethical Committee of Health (REK South-East) and was carried out according to the Declaration of Helsinki’s principle for biomedical research. Written, informed consent was obtained from all donors.

Normal human oral keratinocytes were isolated from mucosal biopsies obtained during third molar extractions. Biopsies were transported in IMDM (Sigma-Aldrich) supplemented with 100 U·mL^−1^ penicillin-streptomycin-fungizone (Lonza, Portsmouth, NH, USA). After overnight enzyme digestion in IMDM supplemented with 2.2 U·m L^−1^ dispase (ThermoFisher) at 4 °C the epithelium was detached and transferred to a new container for mechanical and enzymatic digestion using 10 × trypsin-ethylenediaminetetraacetic acid (EDTA) solution (Sigma-Aldrich) to obtain single-cell suspensions. Trypsin activity was halted with addition of one volume fetal bovine serum (FBS) (Sigma-Aldrich) before the cells were pelleted at 1 000 r·min^−1^ for 5 min. The cells were then resuspended in 5 mL complete K-SFM constituting K-SFM with L-glutamine, 1 ng·mL^−1^ epidermal growth factor, 25 μg·mL^−1^ bovine pituitary extract (both from ThermoFisher) and 100 U·mL^−1^ penicillin-streptomycin-fungizone and evenly distributed in a 25 cm^2^ culture flask (VWR, Radnor, PA, USA). Sub-confluent flasks (~80%) were passaged 1/3 or 1/4 by enzyme detachment using 0.25% trypsin-EDTA solution (Sigma-Aldrich) and two volumes FBS for enzyme inhibition. All incubations were done at 37 °C and 5% CO_2_ saturation and the cultures were used between the third and fifth passage.

The oral squamous cell carcinoma cell line PE/CA-PJ-49 clone E10 (hereafter termed J49; ECACC, Salisbury, UK), from a tongue squamous cell carcinoma in a 57-year old male patient, was grown in complete medium constituting IMDM supplemented with 10% fetal bovine serum (FBS; both from Sigma-Aldrich), 2 mM glutamine (Lonza) and 100 U·mL^−1^ penicillin-streptomycin-fungizon. Cells were detached at ~80% confluence by enzyme detachment using 0.25% trypsin-EDTA solution (Sigma-Aldrich) and two volumes FBS for enzyme inhibition before centrifugation and re-seeding in T75 flasks at a density of 4–7 cells per cm^2^. All incubations were done at 37 °C and 5% CO_2_ saturation.

### Stimulations

For the microarray analysis, J49 cells were harvested and 5 × 10^5^ cells were seeded in T75 flasks and left for overnight incubation. The next day, the cells were washed with PBS to remove unattached cells before fresh culture medium containing 10 μg·mL^−1^ *S. mitis* bacterial lysate was added. The cells were then incubated for 24 h. After incubation, the cells were collected as described above and lysed in RLT buffer (Qiagen, Valencia, CA, USA) supplemented with 1% 2-mercaptoethanol (Qiagen) for RNA extraction.

For analysis of cytokine production and AhR activity by real-time reverse transcription-polymerase chain reaction (RT-PCR), 5 × 10^5^ cells were seeded in six-well plates in complete K-SFM and incubated at 37 °C and 5% CO_2_ saturation. After 4 h all wells were washed with PBS and further incubated overnight in reduced K-SFM. Before stimulation, all wells were washed in PBS and pre-incubated for 1 h in reduced K-SFM with or without 10 μmol·L^−1^ AhR inhibitor CH-223191 (Sigma-Aldrich). After the pre-incubation, the whole volume of media was replaced with 2 mL bacterial suspension (3 × 10^7^ bacteria per mL; multiplicity of infection (MOI) 1:60) with or without inhibitor and incubated for 90 min (real-time RT-PCR) or 6 h (prostaglandin E2 (PGE2) enzyme-linked immunosorbent assay (ELISA)). After stimulation, supernatants were centrifuged at 10 000*g* for 10 min at 4 °C to remove residual bacterial cells and transferred to new tubes before they were frozen at – 20 °C for later use in PGE2 ELISA. Cells were washed in PBS and detached by trypsin-EDTA before they were collected in two volumes FBS. After pelleting at 5 000*g* for 3 min the pellets were disrupted in 350 μL buffer RLT with 1% 2-mercaptoethanol (Sigma-Aldrich) and immediately frozen at −80 °C until further processed.

### Illumina bead array

Genome-wide transcriptional analysis was performed using Illumina bead array (San Diego, CA, USA) technique Norwegian Microarray Consortium in Oslo, Norway (project no.: NMC-OSLO-0182).

Utilizing Illumina TotalPrep-96 RNA Amplification Kit, biotin-labeled cRNA was synthesized from 500 ng total RNA by first- and second-strand reverse transcription follow by *in vitro* transcription of cRNA. RNA quantity was determined using a NanoDrop Spectrophotometer while RNA size and integrity were determined using the Agilent 2100 Bioanalyzer (Agilent Technologies, Santa Clara, CA, USA) and RNA integrity number (RIN) values. Values above 7 were considered to be acceptable. All samples had RIN values above 8. 750 ng of biotin-labeled cRNA was analyzed using a HumanHT-12 v4 Expression BeadChip (Illumina, San Diego, CA, USA), each of the 12 arrays consisting of more than 47 000 50-mer gene-specific bead-linked probes, each bead containing hundreds of thousands of probes of the same sequence. Data from the microarray were filtered and processed using the software programs TIBCO Spotfire (Boston, MA, USA) and Ingenuity pathway analysis (Qiagen).

### RNA extraction and real-time RT-PCR

Total RNA was extracted using the QIAcube and the QIAcube standard RNeasy mini with DNase digestion protocol for the RNeasy mini kit (both from Qiagen). The total RNA was eluted in 30 μL nuclease free H_2_O and the RNA quantity and purity (OD_260_/OD_280_ values) were determined using a NanoDrop Spectrophotometer (NanoDrop Technologies, Wilmington, DE, USA).

Complementary DNA (cDNA) was synthesized using the Reverse Transcription Core Kit (BioNordica, Oslo, Norway) according to the manufacturer’s protocol and the GeneAMP PCR System 9700 (Applied Biosystems, Waltham, MA, USA). The thermocycle program was set to 25 °C for 10 min, 48 °C for 30 min and 95 °C for 5 min and the cDNA was kept at 4 °C until it was diluted in nuclease free H_2_0. Real-time RT-PCR was carried out using predesigned KiCqStart primers (human CYP1A1, CXCL1, CXCL2, CXCL8 and RPS26; all from Sigma-Aldrich), SYBR green (Biotool, Houston, TX, USA) and the Stratagene MX 3005P PCR system (Stratagene, La Jolla, CA, USA). After 40 cycles of amplification the amplicon was verified by one cycle of dissociation analysis by increase in temperature by 1 °C per 30 s from set point temperature to 95 °C. The MxPro software program (Agilent Technologies, Santa Clara, CA, USA) was used for PCR setup and data analysis, before fold change calculations of gene expression relative to housekeeping gene *S26*.

### Immunocytofluorescence

Keratinocytes were grown on glass slides in 24-well plates. After 20 min exposure to *S. mitis* (MOI 1:60) the cells were washed twice in PBS, fixed and permeabilized at −25 °C for 5 min using ice cold methanol:acetone solution (7:3). For blocking, 1% IgG-free bovine serum albumin (Jackson Immunoresearch, Newmarket, UK) in PBS was added and left for 30 min at RT before incubated with polyclonal AhR goat anti-human IgG antibody (Santa Cruz, Dallas, TX, USA, 1:150) for 2 h at 4 °C. After washing twice, Cy2-conjugated donkey anti-goat IgG polyclonal secondary antibody (Jackson, 1:150) was added to each well and left for incubation for 1 h before DAPI was added and the slides were mounted on cover glasses.

### ELISA

Prostaglandin E2 levels in supernatants of oral keratinocytes exposed to *S. mitis* for 6 h were determined using the competitive ELISA Prostaglandin E2 Parameter Assay Kit (R&D Systems, Minneapolis, MN, USA). In brief, cell culture supernatant was added to the supplied plate pre-coated with PGE2-specific mouse monoclonal antibodies. After washing, horseradish peroxidase (HRP)-labeled PGE2 was added to bind the unoccupied antibodies in the wells. Then, the HRP activity was determined after addition of substrate by absorbance at 450 nm and was used to inversely correlate the PGE2 concentration in the experimental sample.

### Statistical analysis

The data were analyzed using one-way analysis of variance for repeated measures followed by Holm–Sidak adjustment for multiple comparisons. To comply with requirements of normality and equality of variance, values obtained for transcription (real-time RT-PCR) were log-transformed before statistical evaluation. The statistical analyses were carried out using SigmaPlot (v13; Systat Software, Chicago, IL, USA).

## Results

### Transcriptional regulator activity induced by *S. mitis*

To study the transcriptional regulation in oral keratinocytes exposed to *S. mitis*, we utilized the Illumina microarray technique for a transcriptome analysis of the oral keratinocyte cell line J49 after 24 h exposure to *S. mitis* bacterial extract. The resulting data set was analyzed using the bioinformatics tool Ingenuity Pathway Analysis. We applied an “upstream regulator analysis” to predict altered activity in transcriptional regulators based on the differential expression of known downstream target genes found in our data set. The analysis was restricted to the two selected categories “transcription regulators” and “ligand-dependent nuclear receptors”, further jointly referred to as transcription factors (TFs), in order to exclude epigenetic regulators (that is, microRNA). We found nine TFs that were predicted to be inhibited and 20 to be activated (Table 1). Among the latter was the AhR. As this is an important TF that is involved in regulation of many relevant biological functions, including immunological and inflammatory responses, cell differentiation, cell adhesion and migration^14^ and in humans is directly linked to and significantly improves wound healing, this was chosen for further study.

Functional analyses of the downstream target genes of the remaining TFs revealed that the inhibited TFs were involved in regulation of different cell death mechanisms while the activated TFs clustered mainly within regulation of metabolic processes (Table 2).

### Exposure to *S. mitis* triggers AhR activation in oral epithelial cells

**Figure 1:**
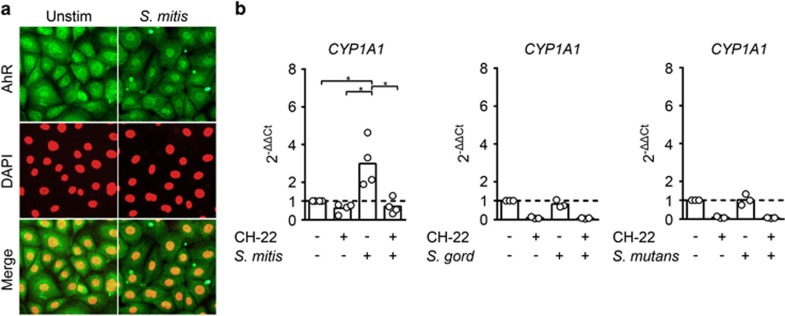
***S. mitis* activates AhR in oral epithelial cells**. (**a**) Nuclear translocation of AhR was examined by immunofluorescence microscopy of human primary oral keratinocytes, cultivated and exposed to live *S. mitis* for 20 min (right) or left untreated (left). Cells were stained for AhR (upper row; green) and DAPI (middle row; red). Merged pictures in the lower row. (**b**) Fold changes in the transcription of *CYP1A1*, the hallmark gene of AhR activation, determined by real-time RT-PCR in human primary oral keratinocytes exposed to live *S. mitis* (left), *S. gordonii* (middle) and *S. mutans* (right) for 90 min, with or without the AhR-specific inhibitor CH-223191 (*n*=3 or 4). Brackets marked with asterisks indicate statistically significance (ANOVA for repeated measures followed by Holm–Sidak adjustment for multiple comparisons; *P*<0.05). ANOVA, analysis of variance.

To verify whether *S. mitis* induces AhR activation, we carried out immunofluorescence microscopy on cultured monolayers of primary human oral keratinocytes and observed increased nuclear translocation of AhR in cultures exposed to *S. mitis* for 20 min as compared with untreated controls (Figure 1a). Next, we analyzed by real-time RT-PCR the regulation of target gene cytochrome P450 1A1 (*CYP1A1*), a hallmark gene for AhR activation,^15^ in oral keratinocytes exposed to *S. mitis* with or without the AhR inhibitor CH-223191. We found that *S. mitis* induced a statistically significant (threefold) increase in *CYP1A1* mRNA levels that were reduced to basal levels when AhR inhibitor was added (Figure 1b). Conversely, in response to *Streptococcus gordonii* and *Streptococcus mutans* the *CYP1A1* mRNA levels remained unaltered (Figure 1b.)

### AhR activation by *S. mitis* increases transcription of *CXCL2* and *CXCL8*

**Figure 2:**
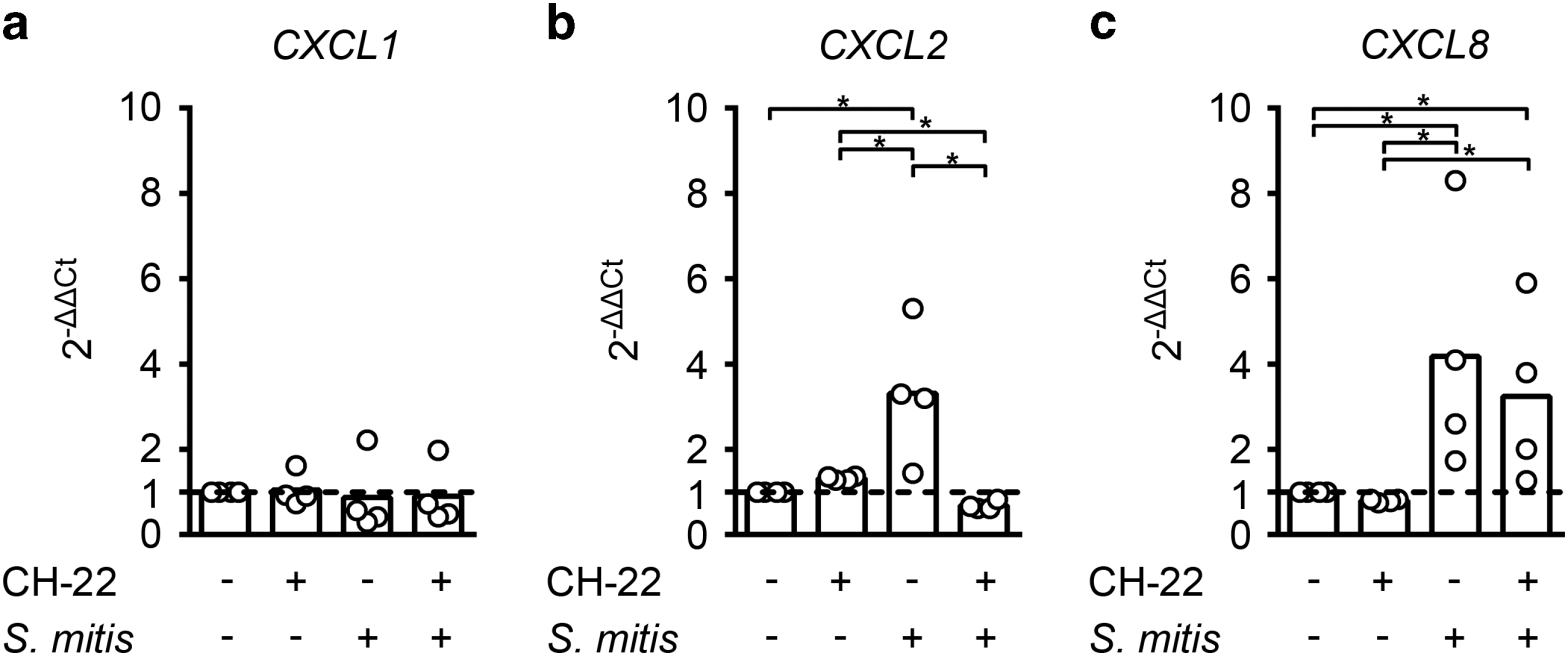
***S. mitis* shows AhR-dependent induction of pro-inflammatory mediators *CXCL2* and *CXCL8*.** Fold changes in the transcription of chemoattractants *CXCL1* (**a**), *CXCL2* (**b**) and *CXCL8* (**c**) were determined by real-time RT-PCR in human primary oral keratinocytes exposed to live *S. mitis* for 90 min, with or without the AhR-specific inhibitor CH-223191 (*n*=4). Brackets marked with asterisks indicate statistically significance (ANOVA for repeated measures followed by Holm–Sidak adjustment for multiple comparisons; *P*<0.05). ANOVA, analysis of variance.

AhR can interact with the NF-kβ subunit RelB and thereby regulate expression levels of *CXCL8*.^16^ We have also shown that *S. mitis* activates transcription of the chemokine *CXCL2* in oral epithelial cell line J49.^13^ To investigate AhR-dependent *S. mitis-*induced regulation of *CXCL1*, *CXCL2* and *CXCL8* mRNA levels, we exposed oral keratinocytes to *S. mitis* with or without AhR inhibitor for 90 min and screened for regulation of the three target genes by real-time RT-PCR. Without inhibitor, *S. mitis* triggered a statistically significant increase of both *CXCL2* and *CXCL8* mRNA levels, three and fourfold, respectively, which could be attenuated by addition of inhibitor (Figure 2b and 2c). *CXCL1* expression levels were not affected by the presence of *S. mitis* (Figure 2a).

### *S. mitis* triggers release of PGE2

**Figure 3:**
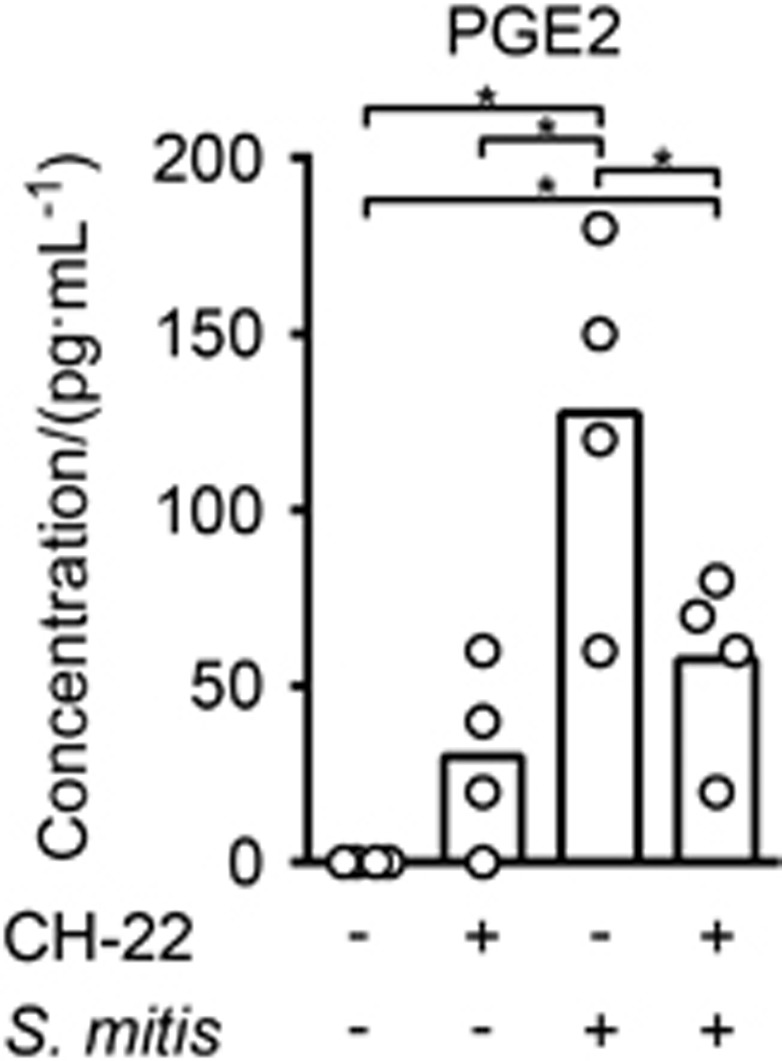
**Oral epithelial cells exposed to *S. mitis* show increased secretion of PGE2.** Human primary oral keratinocytes were exposed for 6 h to live *S. mitis* and/or the AhR-specific inhibitor CH-223191 or left untreated. Supernatants were collected and their PGE2 content was measured by ELISA (*n*=4). Brackets marked with asterisks indicate statistically significance (ANOVA for repeated measures followed by Holm–Sidak adjustment for multiple comparisons; *P*<0.05). ANOVA, analysis of variance.

The differential expression of four highly diverse PGE2 receptors in various types of cells assigns the lipid mediator PGE2 with quite diverse and dose-dependent biological effects.^17^ Human airway epithelial cells exposed to cigarette smoke extracts show an AhR-mediated increase in PGE2 secretion.^18^ We screened the supernatant of oral keratinocytes after 6 h exposure to *S. mitis* using a PGE2 ELISA and found a 127-fold increase in PGE2 levels in response to *S. mitis* that was partially impaired by the AhR inhibitor (Figure 3). The observed differences between the elevated PGE2 levels in response to *S. mitis* relative to unstimulated cells and between the reduced PGE2 levels by AhR inhibitor relative to *S. mitis* alone were both statistically significant (*P*<0.05).

## Discussion

The oral cavity is lined with a multilayered epithelium that protects the host against microbial attack. As the epithelial cells detach from the basal layer and progress outwards, the cells become terminally differentiated, shut down their transcriptional activity and eventually peel off in a process called shedding.^19^ The outermost epithelial cell layers, together with the mucus layer covering them, form an efficient barrier that bar commensals and pathogens access to the deeper epithelial layers and the subjacent tissues and prevent them from entering the mucosae. In case of wounding, however, bacteria can come into contact with epithelial cells in the basal and parabasal cell layers that have the transcriptional capacity to properly respond to the threats. Here, we wanted to study the transcriptional regulation in oral keratinocytes exposed to *S. mitis* to identify potential roles of colonizing *S. mitis* in physiological processes occurring in the oral cavity. An upstream analysis of oral epithelial cells exposed to *S. mitis* revealed altered activity of 29 TFs. A functional analysis of the downstream target genes of these 29 TFs found in our data set was largely grouped in pathways controlling metabolic processes and cell death mechanisms. This is perhaps not very surprising considering the cellular stress inflicted on the cells by exposure to microbes. For further study we chose AhR, a mediator involved in many biological processes including immunological and inflammatory responses, cell differentiation, cell adhesion and migration^14^ and in humans is directly linked to and significantly improves wound healing.^20^

Initially, AhR was characterized by its regulation of detoxifying enzymes in response to environmental toxins, but this is now thought to be a secondary function since AhR is highly conserved throughout evolution and anthropogenic environmental toxins are a relatively new phenomenon.^15^ Activation of AhR can be elicited by both exogenous and endogenous ligands.^15^ In the inactive state, AhR is located in the cytoplasm and upon activation it translocates to the nucleus where it dimerizes with the AhR nuclear translocator and subsequently interacts with the xenobiotic-/dioxin-response element in the promotor region of target genes. This is followed by rapid nuclear export and degradation in the cytoplasm.^21^ Immune staining of oral keratinocytes revealed a nuclear translocation of AhR already after 20 min of exposure to *S. mitis*. The monooxygenase cytochrome P450, family 1, subfamily A, polypeptide 1 (CYP1A1), a member of the cytochrome P450 superfamily^15^ is the hallmark gene upregulated by AhR. In cells exposed to *S. mitis*, CYP1A1 showed a statistically significant (threefold) increase in transcript levels relative to unstimulated control and the responses were silenced by addition of an AhR-specific inhibitor. This shows that *S. mitis* can activate the intracellular receptor AhR and regulate its downstream functions. By contrast, neither *S. gordonii* nor S. *mutans* induced increased transcription of *CYP1A1* after incubation with human primary keratinocytes, indicating that AhR activation is a species-specific characteristic, limited to restricted types of oral commensals, including *S. mitis*.

We next examined the effect of *S. mitis* on the expression of three AhR target genes in oral keratinocytes, *CXCL1*, *CXCL2* and *CXCL8*, chemokines that are important in recruitment of granulocytes and to initiate inflammation. We observed a significant AhR-dependent increase in both *CXCL2* (threefold) and *CXCL8* (fourfold) mRNA levels in cultured oral epithelial cells that dropped to baseline and threefold, respectively, when AhR inhibitor was added. CXCL1 is another chemoattractant that can be regulated by AhR,^22^ but unlike *CXCL2* and *CXCL8*, it remained unchanged in response to after 90 min exposure to *S. mitis*. As the oral keratinocytes used in this study represent cells of the deeper layers of the multilayered oral epithelium, the interaction between *S. mitis* and those keratinocytes is most relevant in case of wounding of the epithelium. When this happens, it is vital that commensals such as *S. mitis* retain the potential to activate host defense mechanisms to favor their uncomplicated elimination. It is indeed a crucial characteristic of commensals that if they step out of their niche they rapidly can be eliminated, a process that is greatly supported by chemokine recruitment of granulocytes.

The most potent known AhR ligand is the halogenated dioxin TCDD (2,3,7,8-tetrachlorodibenzodioxin) while tryptophan^20^ and arachidonic acid (AA) metabolites^15, 23^ are some of the endogenous ligands. Among the AA metabolites, prostaglandins are shown to activate AhR but only at levels exceeding that of physiological concentrations.^24^ Conversely, AhR can modulate inflammation by regulating PGE2 as shown in this study and by others.^18, 25^ PGE2 is not only a mediator of acute inflammation but has also immunosuppressive properties that in the final phases of wound healing contribute to the resolution of inflammation and facilitation of tissue regeneration.^26^ Differential expression of four highly diverse PGE2 receptors with high- and low-affinity receptors in various types of cells can part explain the divergent effects of PGE2.^27, 28^ Gram-negative (G^-^) bacterial species are shown to induce higher amounts of PGE2 compared to Gram-positive (G^+^) species.^29^ Epithelial cells (current study) and monocytes^29^ exposed to G^+^ species *S. mitis* showed PGE2 levels of ~120 and ~1 000 pg·mL^−1^, respectively, but both displayed significantly lower levels compared to G^-^ bacterial species (~5 000 pg·mL^−1^). The currently detected PGE2 secretion was partially regulated by AhR. PGE2 decreases fibroblast proliferation, inhibits collagen synthesis, enhances the expression of MMPs and inhibits myofibroblast differentiation, processes that all must be down-regulated in the remodeling phase of wound healing to avoid scar formation.^17, 26^ Also, exaggerated PGE2 production is associated with enhanced susceptibility to infections.^30, 31^ The moderate AhR-dependent PGE2 secretion in response to *S. mitis* may therefore be another characteristic of *S. mitis* as a commensal that does not induce strong inflammation but favors tight regulation of inflammation, necessary for normal healing processes.^26^

The presently detected effects show that *S. mitis* can modulate inflammatory responses through AhR-mediated regulation of mediators such as chemokines and PGE2. Our findings indicate that *S. mitis* can participate in wound healing in the oral cavity by (i) initial generation of chemokines in order to attract leukocytes to the site of injury and (ii) in the later phases by the release of PGE2 that can participate in the remodeling phase of oral healing. The concept that the oral commensal microbiota can induce AhR activation is important, also in view of the role that AhR has in modulation of T-cell differentiation into Th17 and Treg cells. AhR is also an anti-inflammatory factor in macrophages.^32^

## Figures and Tables

**Table 1:** List of transcriptional regulators with predicted altered activity in response to *S. mitis* lysate.

Upstream regulator	Molecule type	Predicted activation state	Activation *z*-score
Heat shock factor protein 1	Transcription regulator	Activated	3 098
Paired box protein Pax-7	Transcription regulator	Activated	3 030
Hairy/enhancer-of-split related with YRPW motif protein 1	Transcription regulator	Activated	2 586
Myocyte-specific enhancer factor 2C	Transcription regulator	Activated	2 578
Heart- and neural crest derivatives-expressed protein 1	Transcription regulator	Activated	2 449
CCAAT/enhancer-binding protein alpha	Transcription regulator	Activated	2 430
Estrogen receptor beta	Ligand-dependent nuclear receptor	Activated	2 392
Aryl hydrocarbon receptor	Ligand-dependent nuclear receptor	Activated	2 368
Transcription factor Sp3	Transcription regulator	Activated	2 360
Transcription factor Dp-1	Transcription regulator	Activated	2 330
X-box-binding protein 1	Transcription regulator	Activated	2 253
CCAAT/enhancer-binding protein beta	Transcription regulator	Activated	2 224
Krueppel-like factor 1	Transcription regulator	Activated	2 213
Early growth response protein 1	Transcription regulator	Activated	2 197
Peroxisome proliferator-activated receptor gamma coactivator 1-alpha	Transcription regulator	Activated	2 173
Methyl-CpG-binding domain protein 2	Transcription regulator	Activated	2 128
Sterol regulatory element-binding protein 1	Transcription regulator	Activated	2 095
Histone acetyltransferase KAT5	Transcription regulator	Activated	2 025
WW domain-containing transcription regulator protein 1	Transcription regulator	Activated	2 000
Cyclic AMP-responsive element-binding protein 3-like protein 4	Transcription regulator	Activated	2 000
Hepatic leukemia factor	Transcription regulator	Inhibited	−2 000
Transcriptional repressor protein YY1	Transcription regulator	Inhibited	−2 021
COP9 signalosome complex subunit 5	Transcription regulator	Inhibited	−2 155
Transcription regulator protein BACH1	Transcription regulator	Inhibited	−2 172
Nuclear factor 1 C-type	Transcription regulator	Inhibited	−2 236
Death domain-associated protein 6	Transcription regulator	Inhibited	−2 392
Kelch-like ECH-associated protein 1	Transcription regulator	Inhibited	−2 408
Nuclear receptor ROR-alpha	Ligand-dependent nuclear receptor	Inhibited	−2 579
Proto-oncogene c-Rel	Transcription regulator	Inhibited	−2 868

**Table 2:** List of top five biological processes affected by the downstream target genes of the 20 activated (342 target genes) and nine inhibited (95 target genes) transcription factors

	Pathway ID	Pathway description	Count
Activated			
	GO:0010033	Response to organic substance	133
	GO:0031325	Positive regulation of cellular metabolic process	141
	GO:0070887	Cellular response to chemical stimulus	124
	GO:0009893	Positive regulation of metabolic process	148
	GO:0010604	Positive regulation of macromolecule metabolic process	128
Inhibited			
	GO:1901700	Response to oxygen-containing compound	35
	GO:0042981	Regulation of apoptotic process	34
	GO:0010941	Regulation of cell death	34
	GO:0043067	Regulation of programmed cell death	33
	GO:0048583	Regulation of response to stimulus	48
